# Induction of Heat-Shock Protein 70 Expression by Geranylgeranylacetone Shows Cytoprotective Effects in Cardiomyocytes of Mice under Humid Heat Stress

**DOI:** 10.1371/journal.pone.0093536

**Published:** 2014-04-02

**Authors:** Xiaowu Wang, Binbin Yuan, Wenpeng Dong, Bo Yang, Yongchao Yang, Xi Lin, Gu Gong

**Affiliations:** 1 Center of Cardiovascular Surgery, Guangzhou General Hospital of Guangzhou Military Command, Guangzhou, China; 2 Department of Anesthesiology, General Hospital of Chengdu Military Command, Chengdu, China; California State University Fullerton, United States of America

## Abstract

**Background:**

Increasing evidence has revealed that humid heat stress (HHS) causes considerable damage to human health. The cardiovascular system has been suggested to be the primary target of heat stress, which results in serious cardiovascular diseases. However, there is still a lack of effective approaches for the prevention and treatment of cardiovascular diseases induced by HHS.

**Objective:**

Heat-shock proteins (Hsps), especially Hsp70, are reported to provide effective cytoprotection under various stress stimuli. In the present study, we evaluated the cytoprotective effect of geranylgeranylacetone (GGA), which was previously been reported to induce Hsp70 expression in cardiomyocytes under HHS.

**Methods and Principal Findings:**

Using a mouse model of HHS, we showed that the pretreatment of GGA enhanced Hsp70 expression under HHS, as examined by quantitative real-time polymerase chain reaction (qRT-PCR) and Western blot. We then examined the effect of GGA pretreatment on the cardiomyocyte apoptosis induced by HHS using terminal-deoxynucleoitidyl transferase mediated nick end labeling (TUNEL) staining, and found that GGA pretreatment inhibited mitochondria-mediated apoptosis. GGA pretreatment could reverse the effect of HHS on cell apoptosis by increasing expression of Bcl-2, decreasing cytochrome c in cytosol, and increasing cytochrome c in mitochondria. However, GGA pretreatment had no effect on the oxidative stress induced by HHS as determined by levels of superoxide dismutase (SOD), malondialdehyde (MDA), and glutathione (GSH).

**Conclusion:**

We have demonstrated that GGA pretreatment suppressed HHS-induced apoptosis of cardiomyocytes through the induction of Hsp70 overexpression.

## Introduction

Increasing evidence has demonstrated that heat stress leads to considerable damage to human organs, especially the cardiovascular system, which is the primary target of heat stress [1], [2]. Cardiomyocyte apoptosis has been suggested to play a key role in the pathologenesis of cardiovascular diseases including myocardial infarction, cardiac dysfunction, heart failure, and atherosclerosis [2], [3]. Using a mouse model, Qian et al. demonstrated that heat stress caused mitochondrial injury in cardiomyocytes and activated the mitochondria-mediated cell apoptosis pathway [4]. Due to the high energy consumption of the heart, mitochondria occupy up to 30% of the total volume in cardiomyocytes [5]. Mitochondria are vulnerable to cellular stress, such as heat stress [4], and damaged mitochondria have been suggested to be the key mediator of cardiac apoptosis, which releases cytochrome c and excessive reactive oxygen species (ROS) leading to cell apoptosis pathway activation [6]. Therefore, targeting mitochondria-mediated cell apoptosis may be a potential therapeutic strategy for the protection of cardiomyocytes under heat stress.

Exposure to stressful stimuli activates a transient induction of heat-shock proteins (Hsps), which can minimize cellular damage [7]. Hsps are a family of proteins that maintain cellular homeostasis during and after environmental stress such as oxidative stress, chemical stresses, and pathogenic stresses [8], [9]. The family includes a series of different molecular weight members including Hsp105/110, Hsp90, Hsp70, Hsc70, Hsp60, Hsp40, and other low molecular weight Hsps [10]. Different Hsps have different expression profiles in different cells and are localized in different cellular compartments [11]. Among these Hsps, Hsp70 is a well-known member that protects cells and tissues from different pathological conditions [12], [13]. Hsp70 expression is rarely detected in cells under normal growth conditions, but is highly induced under physiological and environmental stress, and is involved in cell protection and tissue repair [14], [15]. It is reported that chronic heat stress significantly elevates the expression of Hsp70 in the rabbit testis, which may play a role in protection from infertility [16]. Hsp70 rescues cells from apoptosis through inhibition of anti-apoptotic proteins such as caspase-3 [17]. Recent studies have revealed that Hsp70 is capable of activating protein kinase B (Akt) to promote cell survival [18]. In rodent cardiomyocytes, Hsp70 plays a role in protection against endotoxemia [19], hypoxia, and metabolic stress [20]. Therefore, the induction of Hsp70 has beneficial effects for cell protection under various stress stimuli.

Whole-body hyperthermia or ischemic preconditioning has been indicated to increase resistance to ischemia-reperfusion injury or damage caused by hepatotoxic compounds through induction of Hsps [13], [21], [22], [23], [24]. However, these strategies cannot be used for clinical manipulation. Pharmaceuticals such as S-nitroso-N-acetylpenicillamine [25], amphetamine [22], and dibutyryl cyclic adenosine monophosphate [26] have been demonstrated to increase resistance to damage partly through the induction of Hsp70 overexpression. Geranylgeranylacetone (GGA), an acyclic polyisoprenoid widely used for ulcer therapy, has been reported to induce Hsp70 expression in cultured gastric mucosal cells [27]. Studies have demonstrated that GGA can directly induce Hsp70 expression in cultured hepatocytes and inhibits cell apoptosis caused by hydrogen peroxide and ethanol [28]. More recently, GGA-induced Hsp70 overexpression has been shown to protect against ultraviolet-induced photokeratitis in mice [29]. However, whether GGA shows protective effects on cardiomyocytes under stress stimuli remains unknown.

In southern China, the climate is hot and rainy with high air humidity, resulting in a humid heat stress (HHS) environment, which might be responsible for the high occurrence of cardiovascular disease [30]. However, little study has been focused on the investigation of cardiac protection under HHS. In the present study, we aimed to investigate the effect of GGA on cardiomyocytes under HHS. Using a mouse model, we showed that GGA pretreatment enhanced Hsp70 expression under HHS. We then examined the effect of GGA pretreatment on the apoptosis of cardiomyocytes induced by HHS and found that GGA pretreatment inhibited mitochondria-mediated cell apoptosis. However, GGA pretreatment had no effect on the oxidative stress induced by HHS. Nonetheless, we showed that GGA pretreatment suppressed the apoptosis of cardiomyocytes through the induction of Hsp70 protein overexpression under HHS. Our study provides evidence that GGA might be used for the prevention and treatment of cardiovascular diseases induced by HHS.

## Materials and Methods

### Ethics statement

This study was carried out in strict accordance with the guidelines of the National Health and Medical Research Council for the Care and Use of Animals for Experimental Purposes in China. The protocol was approved by the Institutional Animal Care and Use Committee of the General Hospital of Chengdu Military Command.

### Experimental animals

Specific pathogen–free (SPF) adult male BALB/c mice (6–8 weeks old, weighing 30–35 g) purchased from the Laboratory Animal Center of Guangdong Province (Foshan, Guangdong, China) were housed under standard conditions of 12/12 h light/dark cycle at room temperature (RT) with routine diet and free access to water in individually ventilated cages (Landbiology, Guangzhou, China). All efforts were made to minimize suffering. For euthanasia, mice were injected with pentobarbital sodium (100 mg/kg, Merck KGaA, Darmstadt, Germany) by intraperitoneal injection before tissue isolation.

### Establishment of humid heat stress animal models and experimental design

To mimic HHS, a hot chamber was used to create an environment with a temperature of 40.0 ± 0.05°C and relative humidity of 60 ± 5%. A total of 32 mice were randomly divided into four groups: (1) eight mice administered vehicle were held at RT (24.0 ± 1°C) with relative humility of 45 ± 5%; (2) eight mice administered vehicle were held at the temperature 40.0 ± 0.05°C with relative humidity of 60 ± 5% for 4 hours per day; (3) eight mice administered GGA (Eisai Co., Ltd, Tokyo, Japan) were held at RT (24.0 ± 1°C) with relative humility of 45 ± 5%; (4) eight mice administered GGA were held at the temperature 40.0 ± 0.05°C with relative humidity of 60 ± 5% for 4 hours per day. Mice were orally administered GGA (500 mg/kg) by using feeding needles, as previously reported [29], 2 hours prior to HHS. The experiment continued for a total of 4 weeks. For experimental analysis, mice were sacrificed by the injection of pentobarbital sodium (100 mg/kg, Merck KGaA, Darmstadt, Germany).

### Quantitative real-time polymerase chain reaction (qRT-PCR) analysis

The RNA of left ventricular tissue was extracted using Unizol Reagent (Biostar, Shanghai, China) according to the manufacturer's instructions. Total RNA (5 μg) was reverse-transcribed into cDNA using M-MLV reverse transcriptase (Clontech, Palo Alto, CA, USA) according to standard protocols. The mixture contained 1 μl of cDNA (1∶50) as template, 5 μl of SsoFast EvaGreen Supermix (BIO-RAD), and 2 μl of each of the forward and reverse primers (1 μM) to a final volume of 10 μl. The PCR procedure was as follows: 94°C for 4 min; 94°C for 20 s, 55°C for 30 s, and 72°C for 20 s; 2 s for plate reading for 35 cycles; and melting curve from 65 to 95°C. GAPDH was used as the internal standard for normalizing gene expression. Three independent experiments were performed. The data obtained were calculated by 2^−ΔΔCt^ and evaluated by statistical analysis as described previously [31], followed by an unpaired sample t-test.

### Western blot analysis

Proteins were extracted from left ventricular tissue and protein concentration was measured by the Bradford method. A total of 20 μg protein was separated by 12% SDS-PAGE electrophoresis followed by electro-blotting onto a nitrocellulose membrane (Amersham, Little Chalfont, UK). Then, the membrane was incubated with 2% non-fat dry milk in Tris-buffered saline (TBS) to block non-specific binding at RT for 1 h. Next, the membrane was incubated with primary antibodies (Santa Cruz, CA, USA) diluted in the blocking buffer overnight at 4°C. Subsequently, the membrane was incubated in horseradish peroxidase (HRP)-conjugated goat anti-rabbit immunoglobulin G (IgG) (Boster Corporation, Wuhan, China) diluted in the blocking buffer for 1 h. 4-Chloro-1-naphthol (4-CN), an HRP substrate, was used for protein visualization.

### TUNEL staining

Left ventricular tissue was isolated from mice and fixed with 4% paraformaldehyde, embedded in paraffin, and cut into 5-μm serial sections. Sections were stained using the TUNEL apoptosis kit (Genmedscientfics, MA, USA) according to standard procedures. Briefly, sections were incubated with TUNEL reaction mixtures for 1 h at 37°C followed by two PBS washes (5 min/time). Then, sections were blocked with glycerol buffer and photographed under a fluorescence microscope (Olympus, Tokyo, Japan). To determine the percentage of apoptotic cells, TUNEL-positive and TUNEL-negative cells were counted using the ImagePro image analysis software (Media Cybernetics, MD, USA). Five fields (500 × 500 μm for each field) per section were randomly selected at ×400 magnification. The analyses were performed in a blinded manner.

### Mitochondrial and cytosolic protein extraction

Mitochondrial and cytosolic fractions were isolated using a mitochondria isolation kit (Pierce, Rockford, USA) according to the manufacturer's instructions. Briefly, mouse left ventricular tissue was isolated and homogenized in ice-cold mitochondria isolation reagent A, and centrifuged at 800 × g for 10 min at 4°C. Sediments were discarded and the supernatants were collected for further centrifugation (12,000 rpm for 20 min at 4°C). Then, the supernatant containing the cytosolic fraction was collected, and the pellet containing the mitochondrial fraction was lysed in 2% CHAPS in Tris-buffered saline (25 mM Tris, 0.15 M NaCl; pH 7.2) for further analysis.

### Caspase-3 activity assay

A total of 20 mg tissue isolated from the left ventricle of mice was homogenized in ice-cold TBS and centrifuged at 12,000 rpm at 4°C for 20 min. The supernatants were collected and incubated with 5 mM Ac-DEVD-MCA at 37°C for 5 min. The release of 7-amino-4-methylcoumarin was measured using a spectrofluorometer (Hitachi F-2000, Tokyo, Japan) using an excitation wavelength of 380 nm and an emission wavelength of 460 nm.

### Determination of antioxidant enzyme activities

Mouse left ventricular tissue was isolated and homogenized in ice-cold physiological saline, and supernatants were collected after centrifugation (10,000 rpm at 4°C). Superoxide dismutase (SOD), malondialdehyde (MDA), and glutathione (GSH) were measured using commercial assay kits (Jiancheng Biotechnology Research Institute, Nanjing, China) as per the manufacturer's instructions. SOD activity was measured using the xanthine oxidase method and absorbance was determined at 500 nm. One SOD activity unit was defined as the amount of enzyme causing 50% inhibition in 1 ml reaction solution/mg tissue protein and the results are expressed as U/mg protein. MDA levels were measured using the thiobarbituric acid method and absorbance was determined at 532 nm. The results are expressed as nmol/mg protein. GSH levels were measured through a reaction using dithiobisnitrobenzoic acid.

### Statistical analysis

All assays were performed in triplicate and data are presented as mean ± standard deviation of the mean (SEM). The statistical significance of differences between two groups was determined by Student t test, and among multiple groups was determined by one-way ANOVA. A p-value of less than 0.05 was considered statistically significant. All statistical analyses were performed using SPSS version 11.5 (SPSS Inc., Chicago, IL, USA).

## Results

### Hsp70 expression is enhanced by pre-administration of GGA under HHS

**Figure 1:**
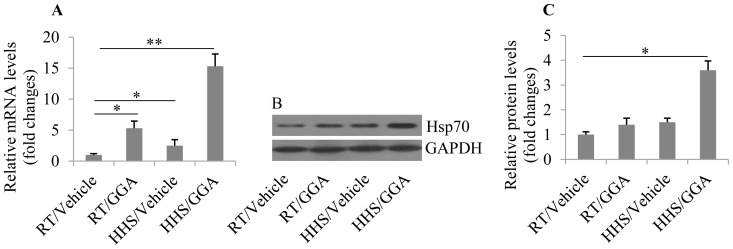
Effect of GGA on Hsp70 expression profiles. Hsp70 mRNA expression (A) and protein expression (B) in cardiomyocytes were analyzed by qRT-PCR and Western blot analysis, respectively. (C) The protein expression was analyzed using Image-Pro Plus 6.0 software and normalized to GAPDH. RT/Vehicle, mice pretreated with vehicle and kept at room temperature acted as controls; RT/GGA, mice pretreated with GGA and kept at room temperature; HHS/Vehicle, mice pretreated with vehicle and treated with HHS; HHS/GGA, mice pretreated with GGA and treated with HHS. *p<0.05 and **p<0.01.

To explore the effect of GGA on Hsp70 expression in cardiomyocytes in mice, we measured the mRNA and protein levels of Hsp70 in mouse left ventricular tissue by qRT-PCR and Western blotting, respectively. The results showed that Hsp70 mRNA levels were increased by ∼5-fold (p = 0.032) compared with control (RT/vehicle) by GGA pretreatment at RT. Under HHS without GGA pretreatment, Hsp70 mRNA expression was slightly upregulated by ∼2.5-fold (p = 0.0248) compared with control. Notably, Hsp70 mRNA expression was dramatically enhanced by GGA pretreatment in the HHS group, by about ∼15-fold (p = 0.0072) compared with control (Fig. 1A). These results suggested that GGA may significantly enhance transcription of the HSP70 gene, especially in the HHS group. These results were further confirmed by Western blot analysis, which demonstrated that Hsp70 protein was abundantly expressed in cardiomyocytes of mice in the HHS group with GGA pretreatment compared with the other groups (p = 0.0182; Fig. 1B).

### Cell apoptosis induced by HHS is inhibited by pre-administration of GGA

**Figure 2:**
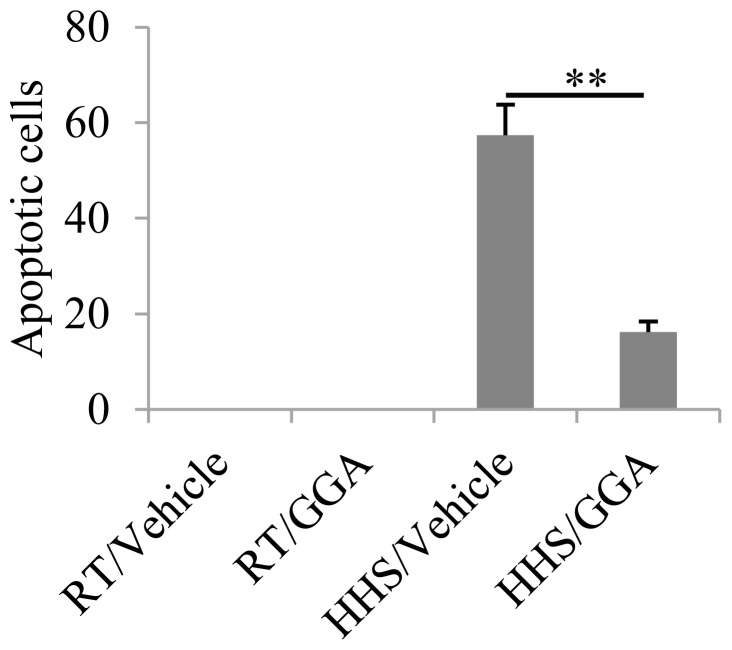
Analysis of apoptotic cells by TUNEL staining. Mean numbers of TUNEL-positive cells in a field of 500 × 500 μm. ** p<0.01.

Next, we investigated whether the overexpression of Hsp70 induced by GGA could regulate cell apoptosis. Using TUNEL staining, we found that the mean number of TUNEL-positive cells in mice was only 16.5 ± 2.2 cells/field in the cardiomyocytes of mice pretreated with GGA in the HHS group, but there were 57.4 ± 6.3 TUNEL-positive cells per field in mice pretreated with vehicle in the HHS group (Fig. 2). The results revealed that the GGA suppressed the apoptosis induced by HHS and that Hsp70 might be involved (p = 0.0034).

### Pre-administration of GGA blocks the mitochondrial apoptotic pathway

**Figure 3:**
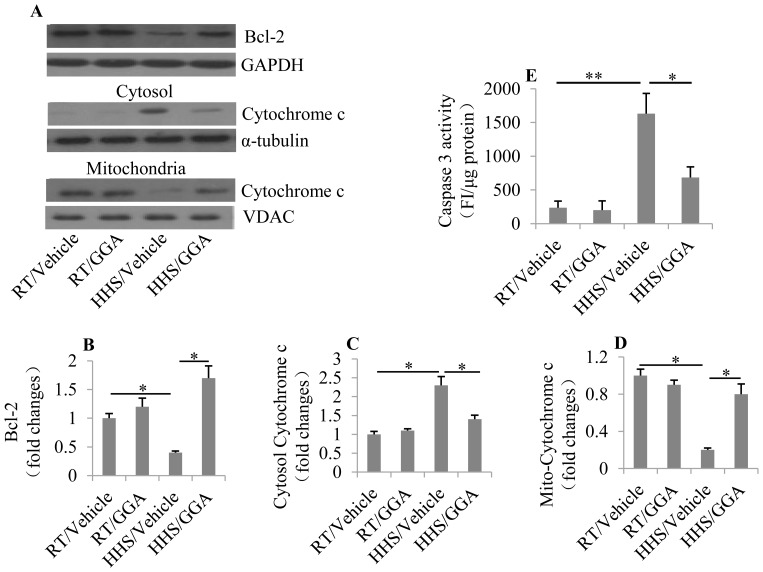
Effect of GGA on the mitochondria-mediated apoptotic pathway. (A) Western blot analysis to examine the protein levels of Bcl-2, and cytochrome c in the cytosol and mitochondria in cardiomyocytes. The protein expression of Bcl-2 (B), cytochrome c in the cytosol (C), and mitochondria (D) was analyzed using Image-Pro Plus 6.0 software and normalized to GAPDH, α-tubulin, and VDAC, respectively. (E) Effect of GGA on caspase-3 activity. A total of 20 mg protein lysate of cardiomyocytes was examined by incubation with Ac-DEVD-MCA for 5 min at 37°C. The release of 7-amino-4-methylcoumarin was measured using a spectrofluorometer. * p<0.05 and ** p<0.01.

Various studies have demonstrated that heat stress activates mitochondria-mediated apoptosis [4], [32]. We examined the genes involved in mitochondria-mediated cell apoptosis including Bcl-2, cytochrome c, and caspase-3 under HHS. The expression of Bcl-2, an anti-apoptotic protein, was significantly reduced in cardiomyocytes under HHS treatment (p = 0.0294); however, it was markedly elevated by GGA pretreatment (p = 0.0138; Fig. 3A and B). Cytochrome c, which is released from mitochondria into the cytoplasm to participate in the formation of the apoptosome, was abundant in the cytoplasm under HHS (p = 0.0223; Fig. 3A and C), implying the activation of apoptosis in cardiomyocytes under HHS stimulation. In addition, GGA pretreatment notably inhibited cytochrome c release from mitochondria (p = 0.0412; Fig. 3A and D). Caspase-3, which is a key downstream effector protein of apoptosis, was also measured. The activity of caspase-3 was significantly increased in the HHS group (p = 0.00414), which could be downregulated by GGA pretreatment (p = 0.0366; Fig. 3E). These data suggest that the overexpression Hsp70 induced by GGA pretreatment protected cardiomyocytes from mitochondria-mediated apoptosis.

### Pre-administration of GGA has no effect on the oxidative stress induced by HHS

**Figure 4:**
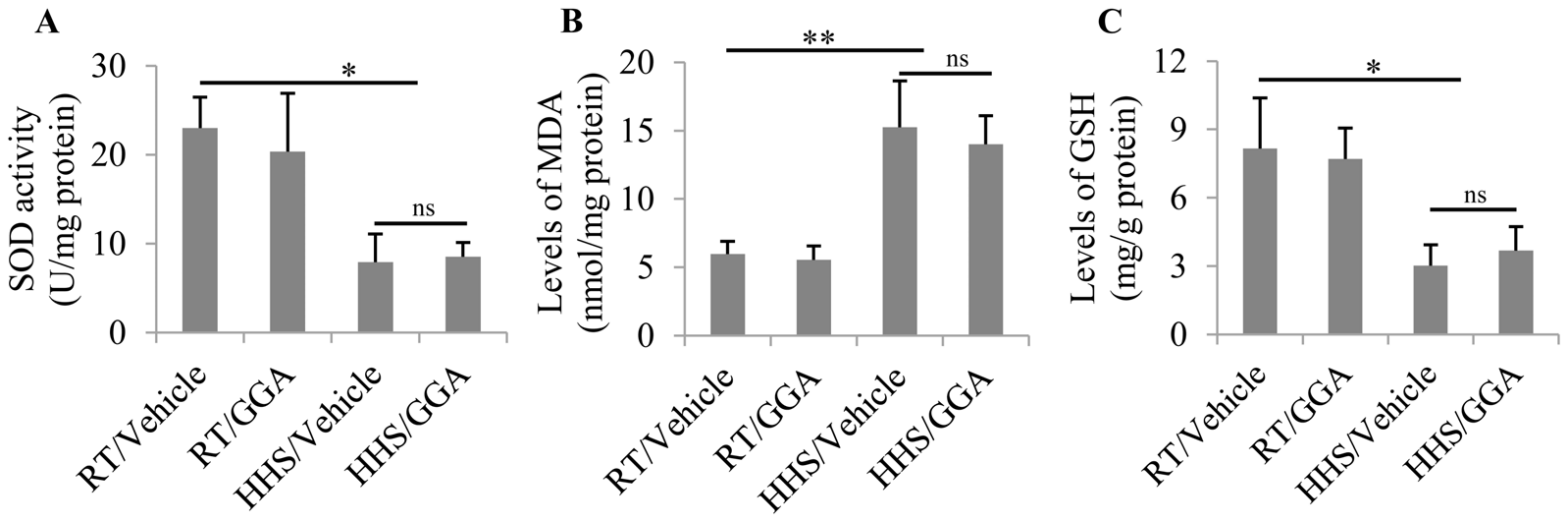
Examination of the effects of GGA on levels of antioxidant and oxidant products. (A) The activity of SOD was determined at 500 nm and the values are expressed as U/mg protein. (B) MDA levels were measured at 532 nm and the results are represented as nmol/mg protein. (C) GSH activity was detected at 420 nm and the data are expressed as mg/g protein. Three independent experiments were performed; * p<0.05 and ** p<0.01; ns indicates no significant difference.

Our previous data have shown that HHS induces oxidative stress, which stimulates cell apoptosis. In order to determine whether the overexpression of Hsp70 has a regulatory effect on oxidative stress, we measured antioxidant and oxidant products. SOD, which was significantly downregulated in the HHS group compared with the RT group (p = 0.0337). However, GGA pretreatment had no effect on SOD levels compared with vehicle pretreatment in the HHS group (p = 0.853; Fig. 4A). MDA, an important marker of lipoperoxidation associated with oxidative stress, was markedly downregulated in the HHS group compared with the RT group (p = 0.015). GGA pretreatment also had no effect on the high level of MDA induced by HHS (p = 1.25; Fig. 4B). Levels of GSH, a common antioxidant, were also significantly reduced in the HHS group (p = 0.0266), but high levels of GSH were not lowered by GGA pretreatment in the HHS group (p = 0.913).

## Discussion

In the present study, we provide evidence that the overexpression of Hsp70 by GGA showed a cytoprotective effect in cardiomyocytes in mice under HHS treatment. GGA-induced Hsp70 mainly protected cardiomyocytes from mitochondria-mediated apoptosis, but had no effect on the oxidative stress induced by HHS. However, continuing efforts should be made to clearly delineate the mechanism of GGA-induced Hsp70 on cellular protection, which may guide the development of new approaches to minimizing the impact of stress stimuli such as HHS.

Cardiomyocytes are regarded to have minimal capacity to undergo apoptosis due to their post-mitotic character, but evidence has accumulated that apoptosis occurs after and during myocardial infarction or coronary arteriosclerosis [3], [33], [34]. Heat stress has also been reported to induce cardiomyocyte apoptosis through action on mitochondria [4]. In accordance with these findings, we demonstrate that HHS induced a high rate of cardiomyocyte apoptosis in the current study. It has demonstrated that heat stress results in the release of proteins located in the mitochondrial inner-membrane space and triggers the activation of the caspase family proteases [4]. Caspase-3 is one of the key mediators of multiple apoptotic signaling pathways including the death-receptor pathway and the mitochondrial pathway [35], [36]. Bcl-2 is an inhibitor of mitochondria-mediated apoptosis [37]. Here, we found that GGA pretreatment significantly decreased cell apoptosis under HHS via increasing Bcl-2 expression and decreasing caspase-3. Previous studies have revealed that GGA induces Hsp70 overexpression and contributed to the inhibition of cell apoptosis under oxidative stress [28], [38]. Hsp70 is a well-known member that protects cells and tissues from different pathological conditions [12], [13]. It has been reported that almost all types of cell stress can induce Hsp expression [39]. Stress stimuli including environment stress and pathological stress commonly cause protein damage, while Hsp70 is the main chaperone responsible for protein folding and the inhibition of protein aggregation [39], [40]. Hsp70 can force target proteins to maintain an adequate structure and retain normal function [41]. It has been reported that Hsp70 overexpression prevents the oxidative-stress-induced decline of mitochondrial permeability transition and the swelling of mitochondria, which may explain the role of Hsp70 in the inhibition of cell apoptosis [42]. Other studies have suggested that Hsp70 suppresses cell apoptosis via blocking JNKs and downstream of JNK activation prior to caspase-3 activation [43], [44], [45]. In the present study, we have demonstrated that GGA pretreatment significantly enhanced Hsp70 expression, which may account for the apoptosis resistance induced by GGA pretreatment under HHS. However, the mechanism of Hsp induction under various stresses remains poorly understood. One of the mechanisms is the activation of heat-shock factor 1 (Hsf-1), which is inactive in the cytoplasm under normal conditions but when activated, binds to Hsps and translocates to the nucleus to enhance Hsp transcription [10], [46]. Ikeyama et al. have described that GGA treatment activates Hsf-1 in mouse hepatocytes exposed to ethanol or H_2_O_2_ [28]. However, further studies should be performed to delineate the molecular mechanism of GGA in the induction of Hsp70 overexpression.

Cell apoptosis is the main form of cardiomyocyte death and increased reactive oxygen species leads to cell apoptosis [47]. ROS are mainly regulated by antioxidant enzymes such as SOD and excessive ROS elicits damage to the cell membrane and causes protein degradation and DNA damage [48], [49]. In the present study, we found that HHS highly decreased the antioxidant enzyme activity, including SOD and GSH activity, and increased the oxidative-stress-associated substance MDA. In contrast, we detected no regulatory effect of Hsp70 expression by GGA pretreatment on the reduction of oxidative stress. In accordance, several studies have also suggested that Hsp70 overexpression reduces nuclear factor (NF)-κB activation, but without affecting ROS production [50], [51], [52]. In addition, Lennikov et al. have reported that GGA-induced Hsp70 overexpression ameliorates UVB-induced corneal damage without ROS suppression [29]. In contrast, a recent study showed that GGA-induced Hsp70 overexpression increased total antioxidant capacity in carbon tetrachloride-exposed adult rat testes [53]. The apparent discrepancy suggests that GGA-induced Hsp70 overexpression might display various functions depending upon the nature of the insult. Interestingly, GGA has recently been reported to attenuate cisplatin-induced reductions in cell viability and caspase-3 activation by suppressing the elevation of intracellular p53 content without heat-shock protein induction [54]. Besides induction of Hsp70, GGA also induces thioredoxin-1, an important modulator of cellular function, which protects cells from various stresses [55], [56], [57], [58]. However, we did not evaluate the effect of GGA on thioredoxin-1 expression under HHS. Whether GGA-induced thioredoxin-1 is involved in the cytoprotection of GGA remains to be further investigated.

In recent years, GGA has been reported as a non-toxic Hsp70 inducer and various studies have suggested that GGA treatment shows cytoprotective effects [27], [28], [38], [59]. There are also other studies demonstrating that other agents such as *Rhodiola rosea* extracts induce Hsp70 expression, which is beneficial for stressed myocardium and biological models of heat shock [60]. More recently, GGA has been found to suppress Alzheimer's disease-related phenotypes, which are similar to those observed in genetically modified mice overexpressing Hsp70 [61]. GGA has been shown to have a protective effect against methamphetamine-induced neurotoxicity in rat pheochromocytoma cells by increasing Hsp70 expression [55]. Our findings further confirm the protective effects of GGA-induced Hsp70 overexpression upon stress stimuli.

Taken together, we demonstrate that oral administration of GGA enhanced Hsp70 overexpression, which was associated with suppressed cell apoptosis, implying a possible application of GGA in the prevention and treatment of cardiovascular disease induced by HHS. However, further studies are necessary to further elucidate the cytoprotective effect of GGA-induced Hsp70 in cardiomyocytes under HHS and the precise underlying molecular mechanism.
